# Professional Incomes in London

**Published:** 1842-10

**Authors:** 


*Professional Incomes in London.*—An interesting Memoir of the late Dr. James Hope, the distinguished author of the Treatise on Diseases of the Heart, has been recently published by Mrs. Hope. The following particulars, in relation to the incomes of the first grade of practitioners in London, will be read with interest:—

On arriving in London, Dr. Hope was led into the belief that the first twenty physicians of the metropolis divided about £80,000 annually between them, and that a successful physician might hope to be established in good practice in about five years. To be one of so large a number as twenty seemed no difficult task, and therefore "he ignorantly hoped" that if he succeeded at all, he should be making £4000 a year in about five years! He was, of course, quickly undeceived. He had now gained many friends, he was married, he was a successul author, but the approaches to £4000 a year were "very tardy." He thought himself in fault, until a little friendly conversation with Dr. Chambers and Sir Henry Halford taught him more correctly upon the subject of so speedily stepping into large receipts. Dr. Hope kept a regular account of every fee which he received during the twelve years he was in London. "We are, therefore," says the Editor, "enabled to speak with the greatest accuracy." The first two years he was in London he made £200 a year. The third year, the accidental removal of some families who employed him, reduced his practice to £150. At the end of the third year his work on the Diseases of the Heart was published, and he came before the profession as a physician to the Marylebone Infirmary. Still, in the first year after, his practice was little increased; but from this period his reputation became extended, and his practice gradually increased, until in eight years more, when he retired, he was making £4000 a year. This—as we should call it—great success was fairly and honorably labored for and won.—*Med. Examiner*, from *Lond. Med. Gaz.*

